# Lapatinib Suppresses HER2-Overexpressed Cholangiocarcinoma and Overcomes ABCB1– Mediated Gemcitabine Chemoresistance

**DOI:** 10.3389/fonc.2022.860339

**Published:** 2022-04-08

**Authors:** Zhiqing Bai, Zhiying Guo, Jiaxing Liu, Yu-Ann Chen, Qian Lu, Ping Zhang, Lili Hong, Yunfang Wang, Jiahong Dong

**Affiliations:** ^1^ Department of Hepatobiliary and Pancreatic Surgery, The First Hospital of Jilin University, Changchun, China; ^2^ Hepatopancreatobiliary Center, Beijing Tsinghua Changgung Hospital, School of Clinical Medicine, Tsinghua University, Beijing, China; ^3^ State Key Laboratory of Bioactive Substance and Function of Natural Medicines, Institute of Materia Medica, Chinese Academy of Medical Sciences and Peking Union Medical College, Beijing, China; ^4^ Institute of Materia Medica, Chinese Academy of Medical Sciences & Peking Union Medical College (State Key Laboratory of Bioactive Substance and Function of Natural Medicines & NHC Key Laboratory of Biosynthesis of Natural Products), Beijing, China

**Keywords:** lapatinib, cholangiocarcinoma, HER2, ABCB1, gemcitabine, chemoresistance

## Abstract

**Background:**

Recent breakthroughs in cholangiocarcinoma (CCA) genomics have led to the discovery of many unique identifying mutations, of which HER2 has been found to be overexpressed specifically in cases of extrahepatic CCA. However, whether or not lapatinib (an oral tyrosine kinase inhibitor selective for inhibition of HER2), or a combination of lapatinib and gemcitabine, exerts inhibitory effects on HER2-overexpressed CCA is still unclear.

**Methods:**

The effect of lapatinib and a lapatinib-gemcitabine combination treatment on CCA was determined using organoid and cell line models. Cell cycle arrest, apoptosis and proteins involving HER2-dependent downstream signaling pathways were analyzed to assess the effect of lapatinib on HER2^+^ CCA. The synergistic effect of lapatinib and gemcitabine was interpreted by docking analysis, ABCB1-associated ATPase assay, rhodamine transport assay and LC-MS/MS analyses.

**Results:**

dFdCTP, the active metabolite of gemcitabine, is proved to be the substrate of ABCB1 by docking analysis and ATPase assay. The upregulation of ABCB1 after gemcitabine treatment accounts for the resistance of gemcitabine. Lapatinib exerts a dual effect on HER2-overexpressed CCA, suppressing the growth of CCA cells by inhibiting HER2 and HER2-dependent downstream signaling pathways while inhibiting ABCB1 transporter function, allowing for the accumulation of active gemcitabine metabolites within cells.

**Conclusions:**

Our data demonstrates that lapatinib can not only inhibit growth of CCA overexpressing HER2, but can also circumvent ABCB1-mediated chemoresistance after gemcitabine treatment. As such, this provides a preclinical rationale basis for further clinical investigation into the effectiveness of a combination treatment of lapatinib with gemcitabine in HER2-overexpressed CCA.

## Introduction

Cholangiocarcinoma (CCA) is the second most commonly occurring hepatobiliary malignancy after hepatocellular carcinoma (HCC) (1). Because most patients with early stage CCA are asymptomatic and often only diagnosed at advanced stages, this results in an overall dismal prognosis (2, 3). CCA can be categorized into two main types: intrahepatic CCA (iCCA), originating within the hepatic parenchyma, and pCCA/dCCA, which can be further classified into either perihilar or distal tumors (4).2’,2’-difluoro-2’-deoxycytidine (gemcitabine), is a fundamental component of the chemotherapeutic agents used in the treatment of CCA. However, gemcitabine-based chemotherapies are limited in their ability to provide therapeutic effects for patients due in part to the presence of complex mechanisms of chemoresistance (MOC) (5–7). ATP-binding cassette (ABC) transporters are one the mechanisms of MOC, actively helping tumor cells export intracellularly active agents across the cell membrane, greatly impairing the cytotoxic effects. Furthermore, the ATP-binding cassette subfamily B member 1 (ABCB1) gene encoded protein ABCB1 (also known as P-glycoprotein or MDR1), has been previously reported to be highly expressed in CCA, is a potential key contributing factor to the degree of drug refractory (8, 9). As such, better strategies for improving the clinical outcomes of gemcitabine treatments and the discovery of novel targeted molecular therapy still urgently need to be developed.

The independent characterization of CCA at the genomic, epigenetic and molecular level helped to ascertain pathogenesis mechanisms, while shedding new light on novel therapeutic options and assisting with precision medicine innovation. One of the most clinically significant breakthroughs of cholangiocarcinoma genomics is the discovery of frequent IDH and FGFR2 mutations in iCCA, the inhibitors of which are currently being evaluated with promising results in clinical trials (10–12). Unlike iCCA, eCCA is found to be more likely to harbor either mutations or amplifications in TP53, HER2/3, ARID1B, etc. (13, 14). When considering all the results from the different studies (**Table S1**), the frequency of HER2 amplification is found to account for approximately 1.3%-23% of all biliary tract carcinoma patient populations. Recent developments in CCA molecular biology has brought more attention to using HER2 as a potential target. An article by Javle et al. (15)reported that a HER2 blockade might be a promising treatment strategy for CCA patients with HER2-overexpression. In the NCT02091141 (“My Pathway”) phase 2 prospective study, 2 of 7 patients with HER2^+^ pCCA/dCCA achieved a partial response after treatment with trastuzumab and pertuzumab (16).

Lapatinib, an oral tyrosine kinase inhibitor selective for inhibition of HER1, HER2, and HER1/HER2-dependent downstream signaling pathways (17), has been widely applied in successfully treating HER2-positive breast, colorectal, and non-small-cell lung cancers (18, 19). Current data from several studies examining the effectiveness of HER2 directed therapy in advanced CCA cases are contradictory and thus, inconclusive. Several of the early phase-2 clinical studies involving use of lapatinib (20) or afatinib (21) have had disappointing results, but these experimental therapies focused mainly on patients harboring EGFR mutations rather than those of HER2. Upon further assessment using immunohistochemical staining, fluorescence *in situ* hybridization and sequencing found that none of the previous cases were those of HER2-overexpression, and therefore it cannot be definitively concluded that lapatinib is ineffective at treating HER2^+^ CCA. We therefore needed to assess whether or not lapatinib can inhibit the growth of HER2^+^ CCA. Moreover, as emerging evidence suggests that many TKIs, including lapatinib have been found to be able to interact with ABCB1 and behave as an ABCB1 inhibitor (22, 23), we believed that TKIs may have important implications in inhibiting ABC transporters and overcoming drug resistance, providing a new opportunity for use in combination with conventional chemotherapies (24). As a result, we investigated the potential synergistic repressive influences lapatinib has on the viability of HER2^+^ CCA cells when combined with gemcitabine.

In this article, we show that gemcitabine upregulates ABCB1 while dFdCTP, an active metabolite of gemcitabine, is the main substrate of ABCB1 and can be expelled from tumor cells. Increasing levels of ABCB1 expression reduce the intracellular drug concentration, resulting in gemcitabine-resistance. Because lapatinib exerts anti-tumor effects on HER2-overexpressed CCA cells while simultaneously overcoming ABCB1–mediated chemoresistance, this proves that a lapatinib-gemcitabine combination-based therapy can be significantly more effective at treating HER2-positive CCA cases.

## Materials and Methods

### Establishment of Organoids Using CCA Specimens Obtained From Patients

For organoid cultures, primary tumor tissues were obtained from patients who underwent radical resection of cholangiocarcinoma, as confirmed by pathological examination at the Beijing Tsinghua Changgung Hospital (Beijing, China). This study was approved by the Ethics Committee of the Beijing Tsinghua Changgung Hospital, and informed consent was obtained prior to surgery. Information about the tumor tissue specimens obtained from patients is stated in **Table S2**.

Fresh tissue samples (not exceeding 4 hours from isolation) were minced into indistinguishable pieces using a surgical scalpel and then incubated in a prewarmed digestion solution at 37°C. The digestion solution is composed of Collagenase IV (Sigma-Aldrich C5138), at the concentration of 1 mg/ml. The overall digestion time lasted no longer than 90 minutes, with extra care being taken not to overdigest the sample. After incubation, cells were filtered through a 70-μm filter and resuspended in Matrigel (Corning 354230) or BME-002 (R&D Biotech) and then carefully administered one droplet at a time on a prewarmed ultra-low attachment cell culture plate (Corning). The culture medium is composed of Advanced DMEM/F12 (Gibco) supplemented with 0.1% Bovine Serum Albumin (Sigma) 1×Glutamax, 10 mM HEPES, 1×penicillin/streptomycin, 1×N2 supplement, 1×B27 supplement, 50 ng/mL recombinant human EGF (all from Life Technologies), 1.25 mM N-acetylcysteine, 10 nM [Leu15]-gastrin I human, 10 mM nicotinamide (all from Sigma), 500 ng/ml R-Spondin 1, 100 ng/ml Wnt3a (all from R&D), 5 µM A83-01, 10 µM forskolin (all from Tocris), 100 ng/ml recombinant human FGF10, 25 ng/ml recombinant human HGF, 25 ng/ml Noggin(all from PeproTech). For the first six days of culture, 10 μM ROCK inhibitor Y-27632 (Tocris) was included. The medium was changed every 2-3 days and cells were then passaged either through mechanical disruption or TrypLE digestion every 10–14 days with a 1:5 split ratio.

### HER2 Expression Evaluated Using Immunohistochemistry and FISH

Immunohistochemical overexpression of HER-2 was evaluated using the Vectastain ABC kit (Vector Labs) following the manufacturer’s protocol and HER2 antibodies (4290, Cell Signaling Technology). Results were interpreted according to current criteria used for gastric cancer (25).Briefly, HER2+++ represents strong complete, basolateral or lateral membranous reactivity in ≥10% of tumor cells; HER2 amplification was evaluated by fluorescence *in situ* hybridization (FISH) probe sets (LBP medicine science and technology) and FISH positivity was defined by the HER2:CEP17 ratio≥2.0 (26).

### cBioPortal

The cBioPortal for Cancer Genomics (http://www.cbioportal.org) provides multidimensional resource of cancer genomics data (27). We investigated the copy number alterations (CNA) in 334 cases with copy number alternation data diagnosed as bile tract carcinoma.

### Cell Lines and Cell Cultures

Three cholangiocarcinoma cell lines (RBE, HUCCT-1 and FRH-0201) were purchased from Yaji Co (Shanghai, China). Breast cancer cell lines were purchased from American Type Culture Collection (ATCC). These cell lines were maintained in Dulbecco’s modified Eagle’s medium supplemented with 10% fetal bovine serum, streptomycin (100 μg/mL) and penicillin (100 U/mL).

### Cell Viability Assay and Drug Combination Study

The cytotoxicity of the drug treatments was evaluated using the CellTiter-Glo Luminescent Cell Viability Assay (Promega) according to manufacturer guidelines. Briefly, cells were seeded in 96-well plates at the appropriate density per well. Cell viability was assessed after being treated with DMSO, lapatinib, gemcitabine, or both drugs in combination for two days. Before the experiment, equilibrate the plate, its contents and celltiter reagent to room temperature. Mix the CellTiter-Glo Buffer with CellTiter-Glo Substrate, Add a volume of it equal to the volume of cell culture medium in each well. Mix, stabilize and record the luminescence. Organoid viability was determined by following a drug-screening method previously established by Hans Clevers (28). Split organoids into single cells 2 days before the start of the screening. Initiate the experiment by filter the organoid using a 70-μm cell strain. Count and resuspend them in organoid medium containing 5% (vol/vol) BME. Dispense these organoids into drug-screening plates and add chemotherapeutics into the culture plate. Organoids will be exposed to drugs for 4 days. The fraction affected by the dose (FA) is defined as the fraction affected by the dose, and combination index CI value was computed using Chou–Talalay means (29), where CI <1, = 1, and >1 indicated synergism, additivity, and antagonism, respectively.

### Establishment of Gemcitabine-Resistant Cells

Initially, FRH-0201 cells were cultured in a 1×IC50 gemcitabine supplemented medium for the duration of 3 days. Once the surviving cells reached an 80% confluency and exhibited stable proliferation after passaging, they were exposed to an increasing gemcitabine concentration gradient of 2 µM, 4 µM, 6 µM, 8 µM until a final concentration 20µM. Gemcitabine-resistant CCA cell line FRH0201-GEM was achieved when cells displayed stable growth and continued passaging in the presence of the drug at 20 μM.

### Western Blotting Analysis

Total protein was obtained by lysing cells with Laemmli Sample Buffer (Biorad) and the membrane protein was extracted according to the manufacturer’s instructions from the Membrane Protein Extraction Kit (Beyotime). Cleared lysates were resolved by SDS-PAGE, transferred to PVDF membrane and probed with specified antibodies. The following primary antibodies were purchased from Cell Signaling Technology: phospho-Akt(4060), Akt(4691), phosphop-p44/42 MAPK(4370), p44/42 MAPK(4695), HER2(4290), phospho-HER2(2249), Cyclin D1(55506), p27 ^Kip1^(3686), c-myc(5605), ABCB1(13978), Na,K-ATPase α1 (23565, control for membrane proteins), and GAPDH(5174, control for total proteins).

### Immunocytofluorescense and Multiplex Immunofluorescence

Tissues and organoids were formalin-fixed, paraffin-embedded and sectioned (4 μm) for histological examination. Sections were first stained with hematoxylin–eosin (H&E). Multiplex immunofluorescence was performed with TSA-dendron-fluorophores (NEON 5-color Allround Discovery Kit for FFPE, Histova Biotechnology, NEFP5100) according to the manufacturer’s protocol. The 4 μm paraffin sections were first deparaffinized, rehydrated, and then the endogenous peroxidase was quenched followed by the addition of a blocking reagent. Then sections were incubated with primary antibodies and corresponding secondary antibodies (Zhongshan Golden Bridge) and stained with TSA-dendron-fluorophores. The primary and secondary antibodies were thoroughly eliminated by microwaving the slides in retrieval/elution buffer. With regard to immunocytofluorescense, cells were fixed after treatment in a 4% formaldehyde and blocked with horse serum. After that, cells were incubated with primary antibody followed by Alexa Fluor 488 conjugated secondary antibody (Thermo Fisher Scientific). Slices were counterstained with DAPI. The following primary antibodies were used: MUC-1 (ab109185), CK7(ab68459) (from Abcam), HER-2 (4290, CST).

### Real-Time Quantitative PCR

The total RNA was extracted from organoids or cell lines using the Trizol^®^ reagent (Invitrogen) and cDNA was synthesized using the ReverTra Ace^®^ qPCR RT Master Mix with gDNA Remover (Toyobo life science). Real-time qPCR was performed using the THUNDERBIRD^®^ SYBR^®^ qPCR Mix (Toyobo life science). Relative gene-expression levels were calculated using the delta–delta CT method. A total of three biological replicates were performed. The primer sequences are as follows: 5’-TTGCTGCTTACATTCAGGTTTCA-3’ and 5’-AGCCTATCTCCTGTCGCATTA-3’ for the ABCB1 gene, 5’-TGTGGGCATCAATGGATTTGG-3’ and 5’-ACACCATGTATTCCGGGTCAAT-3’ for the GAPDH gene.

### Docking Analysis

All docking analyses were performed using MOE software (Molecular Operating Environment, Chemical Computing Group, Montreal, Canada). The ABCB1 protein model was retrieved from the DeepMind algorithm AlphaFold (entry: P08183), based on deep neural network learning (30). The ABCB1 model was then refined through energy minimization, under the parameters set to AMBER10: EHT force field and the active sites of the ABCB1 protein were predicted using the “Site Finder” feature. The structure of dFdC, dFdU, dFdCTP, lapatinib and verapamil were downloaded from the ZINC database (31) and structural refinement and energy minimization were executed. The drugs were docked into the active site of protein using the DOCK module of the MOE algorithm separately (32). Finally, “Triangle Matcher, London dG” and “Induced fit algorithm, GBVI-WSA dG” parameters were chosen respectively as the placement and refinement methods for the docking (33).

### Cell Cycle and Apoptosis Analysis

FRH-0201 cells were seeded at 2×10^5^ cells in a 6 cm cell culture dish and cultured overnight, after which the cells were exposed to one of three different treatments: varying concentrations of lapatinib(0, 1, 5, 20 μM) at 48 hours, constant concentration exposure (20 μM) for different time durations (0, 12, 24, 48 hours) and a combination of a varying concentrations of lapatinib(0, 1, 5, 10 μM) and a constant concentration of gemcitabine(0.5 μM) over a duration of 48 hours. The cell cycle distribution was detected by staining DNA with propidium iodide (C1052 Beyotime), while apoptosis and necrosis were detected using an Annexin V-FITC staining kit (C1062 Beyotime). The cell cycle distribution and apoptosis were determined through flow cytometry (Beckman CytoFLEX).

### Rhodamine Transport Assay

The organoids or cell lines were incubated with 2 μM Rhodamine 123 (MedChemExpress) at 37°C for 5 minutes and washed three times with the culture medium. Afterwards, cells were incubated in the culture medium with varying lapatinib concentrations at 37°C for 30 minutes. The cell lines were then analyzed by flow cytometry (Beckman CytoFLEX). The immunofluorescence of organoids was visualized using a confocal microscope (Olympus FV3000).

### ATPase Assay

The ABCB1-associated ATPase activity was measured using the Pgp-Glo™ Assay Systems (Promega). Briefly, ABCB1 membranes were incubated with gradient concentrations of verapamil, lapatinib or dFdCTP on the 37°C heat block for 5 minutes. Then 10 μl of 25 mM MgATP were added into each well and incubated at 37°C for 100 minutes. Then the reaction was terminated by adding 50 μl ATP Detection Reagent into all wells. Luminescence was then read on a multi-detection microplate reader (Biotek).

### LC-MS/MS Analysis

FRH-0201 and FRH0201-Gem cells were treated with a combination of a varying concentrations of lapatinib (0, 1, 5, 20 μM) and a constant concentration of gemcitabine (1 μM). Cells treated with 1 μM Gem + 5 μM Verapamil were set as the positive control. Cells were exposed to these drugs for 6 hours. To determine the concentration of intracellular dFdCTP, we employed LC-MS/MS. The cells were collected with 0.05% Trypsin-EDTA, and washed three times with cold PBS. Then the cells were counted and collected into 1.5 ml centrifuge tubes. Intracellular fluid was extracted by using a MeOH/H_2_O (4:1) solvent. The standard linear calibration curve was established by diluting a 10 μg/mL standard stock solution into a concentration gradient. We performed LC-MS/MS analysis using AB Sciex Exion LCTMAD chromatography coupled with an electrospray ion source and AB Sciex QTRAP 6500+ mass spectrometry (AB SCIEX, USA). Samples were loaded onto an ACQUITY UPLC HSS T3 column (2.1×100mm, 1.7μm, Waters, USA) and eluted using a solution of 30 mM formic acid (phase A)/acetonitrile containing 0.1% formic acid (phase B). The LC parameters were set as follows: column temperature, 50°C; flow rate, 0.35mL/min; injection volume, 2 μL. The dFdCTP was gradient eluted with phase A/phase B. Mass spectrometry data was acquired on the multiple reaction monitoring (MRM) positive mode, with the ion source temperature set at 550°C, ion source voltage at 4500, curtain gas as 35 psi, gas1 as 60 psi, gas2 as 60 psi, MRM transition as 504.2/326.4, declustering potential (DP) as 63V and collision energy as 31eV. Peak processing and integration were completed using SCIEX OS software (AB SCIEX).

### Statistical Analysis

All statistic data were presented as the mean ± standard deviation (SD) of at least three independent experiments, and statistical analyses were performed with GraphPad Prism version 7.0. Significance was denoted as n.s. not significant, *p < 0.05, **p < 0.01, and ***p <0.001.

## Results

### HER2 Amplification Is Related to Dismal Prognosis in Biliary Tract Carcinoma

In order to investigate the amplification status of HER2 in patients with biliary tract carcinoma, HER2 copy number alternations (CNAs) in biliary tract cancer populations were analyzed, including a total of 329 patients CNA data using the cBioPortal tools from 3 studies: Gallbladder Cancer (MSK, Cancer 2018), Cholangiocarcinoma (MSK, Clin Cancer Res 2018) and Cholangiocarcinoma (TCGA, Firehose Legacy) (**Figure S1A**). HER2 amplification was found in 14 out of 329 patients, accounting for 4% of total sample size and patients with HER2 amplification were found to have markedly shorter survival times than those with lower levels using a Kaplan-Meier plotter analysis (**Figure S1B**).

### Patient-Derived CCA Organoids With HER2 Overexpression Are More Sensitive to Lapatinib

**Figure 1:**
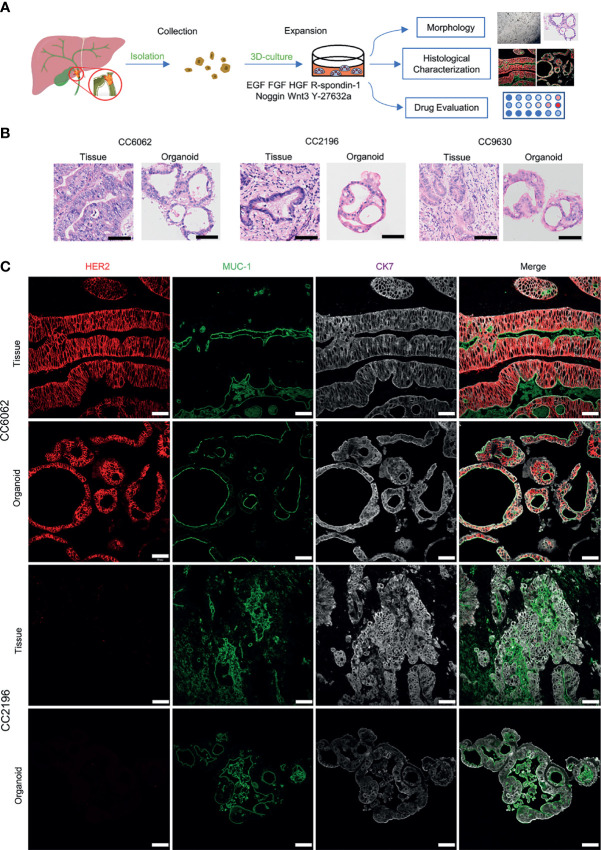
Patient-derived CCA organoids recapitulate the histopathological features of the primary tumors. **(A)** A schematic representation of CCA organoids collection, processing and experimental designs. **(B)** H&E staining images of the CCA tissue specimens CC6062, CC2196, CC9630 and the derived organoids. Scale bar, 50 μm. **(C)** Multiplex immunofluorescence co-staining images of HER2 (red), MUC-1 (green), CK7 (grey) of primary clinical tissue (top row of each group) and organoids (bottom row of each group). Scale bar, 50 μm.

**Figure 2:**
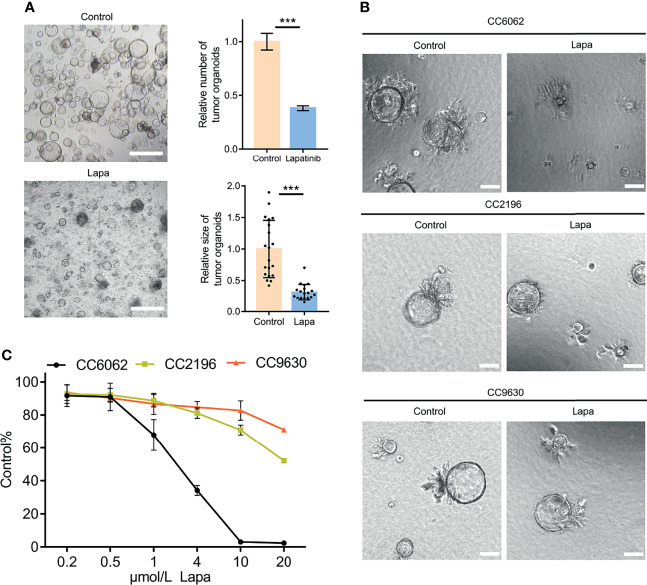
Lapatinib exhibits a stronger inhibitory effect on the growth of HER2-overexpressed CCA patient-derived organoids. **(A)** Bright-field images of HER2-overexpressed CCA organoids treated with 5 μM lapatinib. Relative numbers (n = 3 biologically independent samples per group) and sizes (n =20 biologically independent organoids per group) of organoids were quantified as fold-change compared to control. Scale bar = 200 μm. **(B)** Bright-field images of CC6062, CC2196 and CC9630 treated with 5μM lapatinib (Lapa) compared to control. Scale bar, 20μm. **(C)** Growth inhibitory effect curves of lapatinib (Lapa) in patient-derived CCA organoids proved that CC6062 is more sensitive to lapatinib when compared to other CCA organoids. The data is expressed as the mean ± S.D., two-sided Student’s t-test, ***p < 0.001.

We obtained CCA tissue from patients who underwent radical resection of cholangiocarcinoma with informed consent. We acquired isolated CCA cells from tissue through mechanical disruption and collagenase digestion. Isolated cells were then embedded in Matrigel or BME drops and overlaid with CCA organoid culture medium (reference Methods for more detailed information). We successfully established 3 patient-derived organoid lines of CCA for stable, long-term proliferation (**Figure 1A**). Tissue of CC6062 was proved to be HER2-overexpressed (**Figures S1C, D**), while CC2196 and CC9630 was proved to be HER2-negative (**Figure 1C**). All the H&E-stained primary tissues were found to be that of adenocarcinoma with glandular and tubular structures surrounded by mesenchymal and inflammatory cells, while the organoids established from these tissues were found to be exclusively epithelial tissues with remarkably well-preserved tumor cell organization (**Figure 1B**). MUC-1 and CK7 were the classical molecular markers of CCA (34, 35). The primary tissue and organoids showed similar MUC-1 and CK7 staining patterns (**Figure 1C**), demonstrating that CCA organoids are able adequately to retain their primary tissue histopathological features. Additionally, we found that the original HER2 status of CCA was well maintained in organoid culture, as determined through immunofluorescence staining (**Figure 1C**). Because HER2 status was well retained in the patient-derived tumor organoids, we decided to further examine whether or not lapatinib exerts its growth inhibitory effects on HER2-overexpressed organoids. As shown in **Figure 2A**, both numbers and sizes of CC6062 organoids were drastically reduced after being treated by lapatinib. In contrast, treatment with lapatinib did not result in significant changes in CC2196 and CC9630 (**Figures 2B, C** and **Videos S1A, B, E-H**). Since it has been previously established that lapatinib inhibits both EGFR and HER2, we next examined the EGFR status of these 3 organoid lines. As shown in **Figure S4A**, both the tissue and organoid samples of the lapatinib-sensitive CC6062 were found to be EGFR negative, while those of CC2196 was proved to be EGFR-positive. This proves that lapatinib suppresses the growth of CCA organoids, most likely due to the blockade of the HER2 pathway.

### CCA Cell Line With HER2 Overexpression Is More Sensitive to Lapatinib

**Figure 3:**
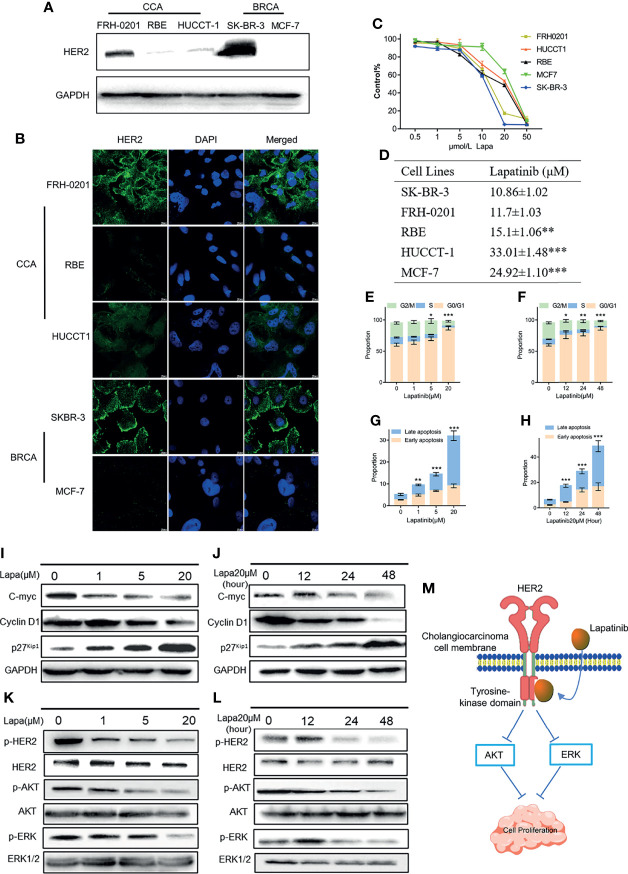
The underlying mechanisms by which lapatinib (Lapa) induces FRH-0201 cell cycle arrest and apoptosis in a concentration- and time-dependent manner. **(A, B)** HER2 protein expression in CCA cell lines was detected by Western blotting and immunofluorescence. **(C, D)** Growth inhibitory effects of lapatinib (Lapa) in HER2^+^ and HER2^-^ cell lines. **(E, F)** Lapatinib dramatically induced G1 cell cycle arrest in HER2-positive CCA cells in both time and dose-dependent manners. **(G, H)** Apoptosis of FRH-0201 was induced post-exposure to varying treatment concentrations of lapatinib or a constant concentration exposure over different time durations. **(I, J)** Down-regulation of c-myc was observed with induction of p27^Kip1^ and down-regulation of Cyclin D1 after lapatinib treatment **(K, L)** HER2 and HER2-dependent downstream signaling pathways were suppressed by lapatinib in FRH-0201 cells. **(M)** A Schematic representation of the mechanisms with which lapatinib inhibits the growth of HER2^+^ CCA cells. The data is expressed as the mean ± S.D., two-sided Student’s t-test, *p < 0.05, **p < 0.01, ***p < 0.001.

Next, we examined the expression level of HER2 proteins in 3 different CCA cell lines. Two breast cancer cells (BCRA) whose HER2 protein expression levels have already been well investigated were set as the positive and negative controls: SK-BR-3 was used as the HER2 overexpression control and MCF-7 was set as the negative control. Both western blotting and immunofluorescence results suggest that the HER2 protein expression in FRH-0201 is very similar to that of SK-BR-3, but RBE and HUCCT-1 are not (**Figures 3A, B**). We examined the inhibitory effect of lapatinib on these five cell lines and the results show that lapatinib was able to suppress cell growth in a concentration-dependent manner more effectively in HER2-overexpressed CCA cell line FRH-0201, than in RBE and HUCCT-1 (**Figure 3C**). In addition, IC50 values of lapatinib in FRH-0201 were found to be on the same level as those in SK-BR-3 (*p* > 0.05). However, in RBE and HUCCT-1 these were significantly higher (*p <*0.001), suggesting that HER2-overexpressed CCA cells are more sensitive to lapatinib (**Figure 3D**). At the same time, we assessed the EGFR status of FRH-0201, RBE and HUCCT-1 (**Figure S4B**). Both FRH-0201 and HUCCT-1 has higher EGFR expression. We used cetuximab (CTX), an EGFR inhibitor, in a side by side comparison. As shown in **Figure S4C**, 1μM CTX can inhibit the phosphorylation level of EGFR, however, this concentration of CTX has no inhibitory effect on FRH-0201(**Figure S4D**). This suggests that the inhibitory effect of lapatinib on FRH-0201, a cell line overexpressing both EGFR and HER2, is mainly achieved by inhibiting the HER2 pathway rather than the EGFR pathway.

### Lapatinib Induces Cell Cycle Arrest and Apoptosis in FRH-0201 in a Concentration and Time- Dependent Manner

Having determined that lapatinib has a robust inhibitory effect on HER2-overexpressed organoids and cells lines, we analyzed its effects on the cell cycle and apoptosis in order to identify the underlying mechanism responsible for this inhibition. FRH-0201 was treated with lapatinib at the previously indicated concentration gradient (0, 1, 5 and 20μM) during a set period of 48 hours and at 20 μM in varying time durations (0,12, 24 and 48 hours). The percentage of FRH-0201 cells arrested in the G1 phase was significantly increased after being treated with lapatinib in a time and concentration-dependent manner (**Figures 3E, F** and **Figures S2A, C**). A down-regulation of c-myc was observed, consistent with the suppression of cyclin D1 and the induction of p27^Kip1^ as determined by Western blot analysis (**Figures 3I, J**). Meanwhile, apoptotic cells increased drastically after being treated with lapatinib with increasing dose and time (**Figures 3G, H** and **Figures S2A, C**). HER2 and HER2-dependent downstream signaling pathways were inhibited after the treatment with lapatinib, as shown by a decreased level of phosphorylation of HER2 and its downstream proteins (**Figures 3K, L**). In conclusion, lapatinib results in definite reduction of p-HER2, p-ERK and p-Akt in HER2-overexpressed cholangiocarcinoma, and thus induces the G1 cell cycle arrest and apoptosis in a concentration and time-dependent manner (**Figure 3M**).

### Lapatinib and Gemcitabine Synergistically Inhibit the Proliferation of HER2^+^ CCA Cells *In- Vitro*

**Figure 4:**
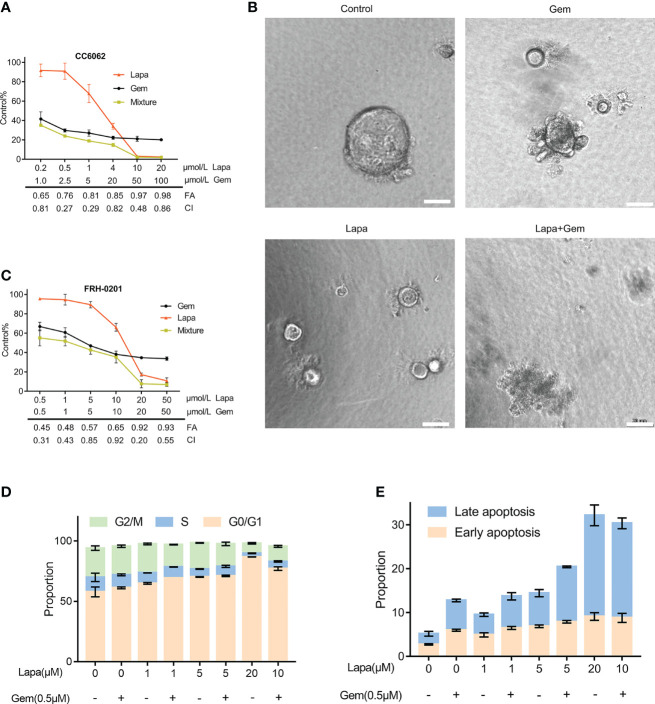
Lapatinib exerts synergistic growth inhibitory effects on HER2-overpressed CCA organoid and cell line when combined with gemcitabine. **(A)** Synergistic growth inhibitory effects of a lapatinib (Lapa) - gemcitabine (Gem) combination treatment of HER2-overexpressed organoids. **(B)** Bright-field images of HER2-overexpressed tumor organoids treated with DMSO, gemcitabine (5 μM Gem), lapatinib (5 μM Lapa) and gemcitabine+lapatinib(5 μM Gem +5 μM Lapa) for 4 days. Scale bar, 20 μm. **(C)** Synergistic growth inhibitory effects of lapatinib (Lapa) combined with gemcitabine (Gem) in FRH-0201. **(D, E)** The lapatinib-gemcitabine combined treatment induced G1 arrest and apoptosis in FRH-0201. Data is expressed as the mean ± S.D. based on three independent experiments.

Because gemcitabine is one of the most significant first-line chemotherapeutic agents of CCA, we considered whether or not lapatinib has a synergistic repressive influence on the viability of HER2^+^ CCA cells when combined with gemcitabine. The efficacy of various concentrations of the combination of lapatinib and gemcitabine was assessed by determining the combination index (CI) which was calculated using the Chou -Talalay method. We observed that lapatinib and gemcitabine dual treatment resulted in stronger growth inhibition in FRH-0201 and CC6062 cells, and that the resulting CI values were consistently less than 1, suggesting that the combination of Lapa and Gem had an overall synergistic effect (**Figures 4A, C**). As shown in **Figure 4B** and **Videos S1A-D**, combination therapy led to higher inhibition of cell growth in CC6062. Further analysis revealed that the percentage of apoptotic and G1 arrested FRH-0201 cells were significantly increased in response to the combined treatments when compared with individual treatments with an increasing lapatinib concentration gradients. In particular, the proportion of apoptotic and G1 arrested cells treated with 10.0μM Lapa +0.5μM Gem 0.5μM corresponded the most to that of cells treated with 20μM Lapa (**Figures 4D, E** and **Figures S2A, B**). In other words, the lapatinib-gemcitabine combined treatment demonstrated a more robust anti-tumor effect in comparison with that of separate treatments.

### Upregulation of ABCB1 Results in Chemoresistance in CCA After Gemcitabine Treatment

**Figure 5:**
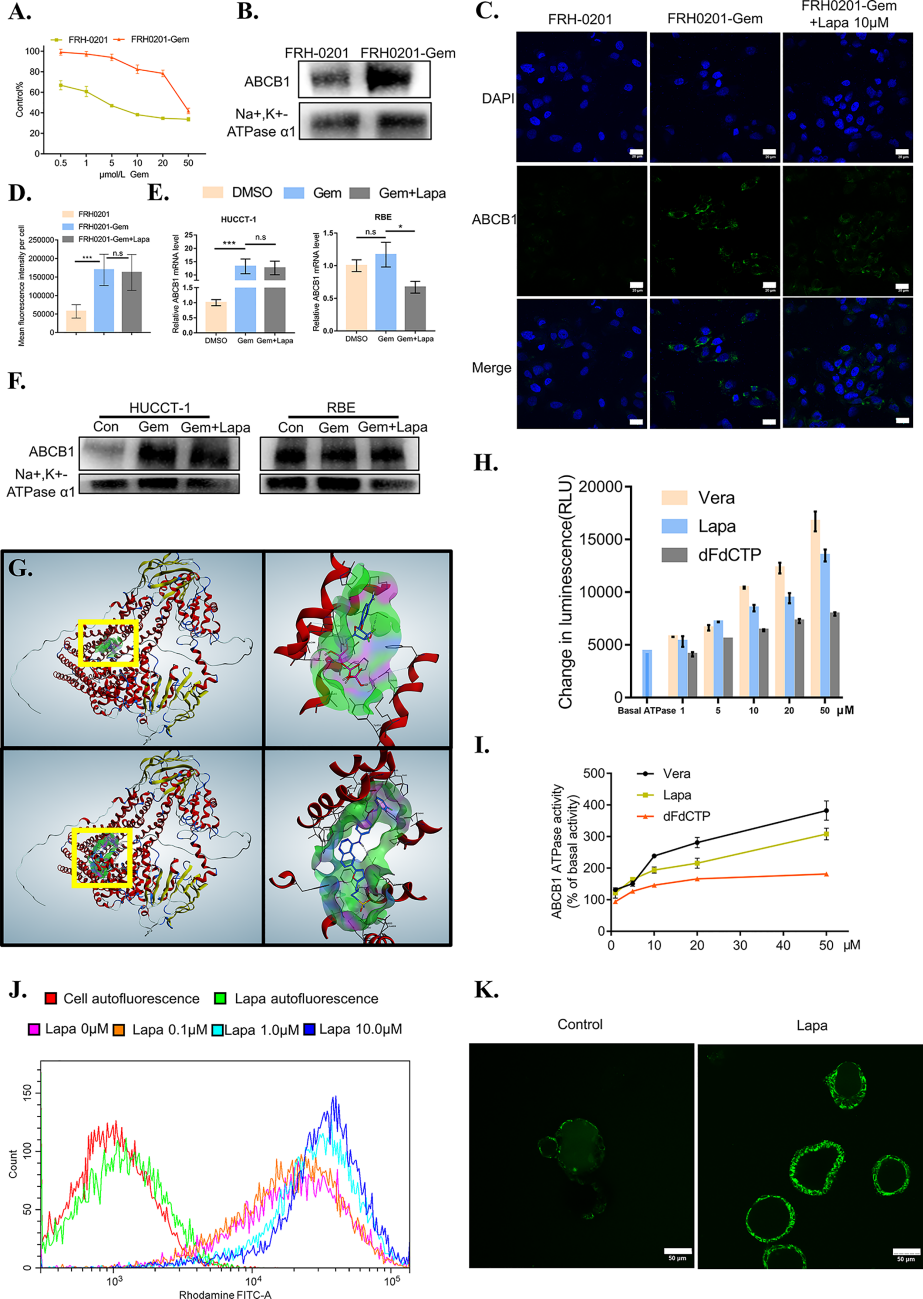
Lapatinib suppresses the function of elevated ABCB1 after being treated with gemcitabine in CCA cells. **(A)** The growth inhibitory effects of gemcitabine (Gem) on FRH-0201 and FRH0201-Gem. **(B, C)** ABCB1 protein expression in FRH-0201 and FRH0201-Gem was detected with Western blotting and immunofluorescence. Scale bar: 20 μm. **(D)** Quantification of fluorescence intensity for ABCB1 in FRH-0201, FRH0201-Gem and FRH0201-Gem incubated with 10μM lapatinib (Lapa) **(E, F)** Relative mRNA levels and protein levels of ABCB1 in various CCA cell lines after treatment with gemcitabine (10 μM Gem) or a combination of gemcitabine and lapatinib (10μM Gem +5μM Lapa) for 48 h. **(G)** Upper left: The panoramic structure of ABCB1 and dFdCTP binding site. Upper right: A detailed three-dimensional plot of the interaction of dFdCTP and ABCB1. Bottom left: The panoramic structure of ABCB1 and lapatinib binding site. Bottom right: A detailed three-dimension plot of the interaction between lapatinib and ABCB1. The ABCB1 protein is depicted in red. H-bondings are shown in purple, hydrophobic bonds are shown in green, and mild polar bonds are shown in blue. Lapatinib and dFdCTP are depicted with the following color codes: carbon (blue), oxygen (red), nitrogen (dark blue), sulfur (yellow), fluoride (green), hydrogen (grey), chlorine (dark green), phosphorus (purple). **(H)** The luminescence increases in a dose-dependent manner following the incubation of verapamil (positive control), lapatinib or dFdCTP with P-glycoprotein-containing membranes. **(I)** ABCB1 ATPase activity increases in a dose-dependent manner with varying concentrations of verapamil, lapatinib, and dFdCTP. **(J)** The fluorescence intensity changes in rhodamine-dyed FRH0201-Gem after treatment with previously indicated concentrations of lapatinib. **(K)** Confocal images of CC6062 dyed with rhodamine after being treated with or without lapatinib. The data is expressed as the mean ± S.D., two-sided Student’s t-test, n.s. not significant, *p < 0.05, ***p < 0.001.

Next, we investigated the mechanism by which lapatinib synergistically inhibits the growth of CCA cells when combined with gemcitabine. Given that ABCB1 is one of the most important, complex mechanisms of chemoresistance (MOC), and many other chemotherapeutics have been proved to increase the expression of ABCB1 (36–41), we considered whether or not gemcitabine could cause the upregulation of ABCB1 and what specific effects lapatinib exerts on ABCB1. We initially established the gemcitabine-resistant CCA cell line, FRH0201-Gem. When we tested the cytotoxicity of gemcitabine comparing the primary and resistant cell lines, higher cellular viability was confirmed in FRH0201-Gem (**Figure 5A**). We can observe the different morphology between FRH-0201 and FRH0201-Gem (**Figure S5A**). Additionally, in the colony-forming assay, after treatment of 5μM gemcitabine, the colony sizes and number of colonies of FRH0201-Gem were larger in comparison with that of the parental FRH-0201, thus demonstrating that FRH0201-Gem cells possess the ability to proliferate for long periods while under continuous exposure to gemcitabine (**Figures S5B, C**). Both western blot and immunofluorescence images suggest that ABCB1 expression was conspicuously elevated in the gemcitabine-resistant CCA cells (**Figures 5B–D**). Even after being treated with gemcitabine, the RNA and protein levels of ABCB1 also significantly increased in HUCCT-1 cells, while gemcitabine-only -treated RBE cells experienced only slight changes (**Figures 5E, F**). This suggests that the upregulation of ABCB1 mediated by gemcitabine might be a commonly occurring phenomenon in CCA.

It is possible that lapatinib’s synergistic effect with gemcitabine can either be achieved through decreasing ABCB1 expression or inhibiting ABCB1 function. Therefore, we first evaluated the effect of lapatinib on the expression of ABCB1. Lapatinib did not decrease the expression level of ABCB1 in FRH0201-Gem as shown in **Figure 5C**. At the same time, in both HUCCT-1 and RBE cells, co-incubation with gemcitabine and lapatinib for a duration of 48 hours resulted in a slight downregulation of ABCB1 at the RNA level, but resulted in no significant changes at the protein level. This proves that the synergistic effect of lapatinib and gemcitabine is not related to the inhibition of ABCB1 protein expression by lapatinib, but instead may be related to the inhibition of ABCB1 function by lapatinib (**Figures 5C–F**).

### Lapatinib Inhibits the Function of ABCB1 Transporters

**Figure 6:**
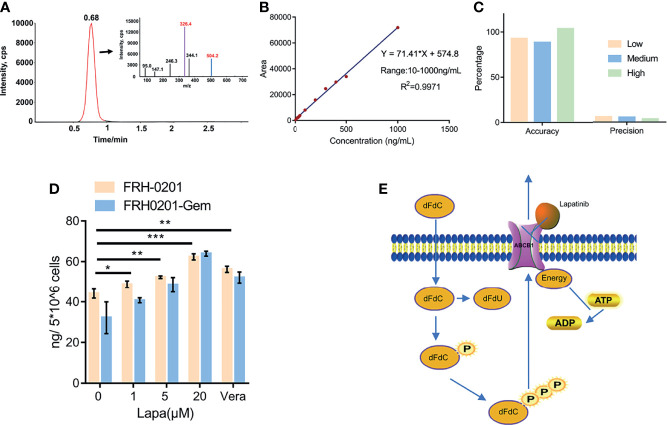
Lapatinib promotes the accumulation of dFdCTP within CCA cells. **(A)** Extracted ion chromatography (XIC) and MS/MS spectrum of dFdCTP. **(B)** Linearity and range of the calibration curve **(C)** Accuracy and precision of the qualitative method under low, medium and high concentrations. **(D)** The concentration of intracellular dFdCTP is positively correlated with increasing concentrations of lapatinib. FRH-0201, FRH0201-Gem were treated with a combination of a varying concentrations of lapatinib (0, 1, 5, 20 μM) and a constant concentration of gemcitabine (1 μM). Cells treated with 1 μM Gem + 5 μM Verapamil were set as the positive control. **(E)** A Schematic representation of the mechanisms with which lapatinib inhibits the efflux of dFdCTP within CCA cells. The data is expressed as the mean ± S.D., two-sided Student’s t-test, *p < 0.05, **p < 0.01, ***p < 0.001.

Since the gemcitabine-only treatment was found to increase the expression of ABCB1 while lapatinib on its own did not influence it significantly, we considered the possibility of whether or not gemcitabine or its metabolites were the substrate of ABCB1 and lapatinib could functionally inhibit ABCB1. As shown in **Figure 6E**, gemcitabine undergoes complex intracellular conversion to nucleotides gemcitabine triphosphate (dFdCTP) (mainly responsible for its cytotoxic actions) and its deaminated metabolite, 2’,2’-difluorodeoxyuridine (dFdU). Initially, we performed a docking simulation analysis in order to predict and evaluate the binding affinities of metabolites of gemcitabine and lapatinib with an ABCB1 model. Verapamil, a classic ABCB1 substrate and inhibitor, with the docking score of –8.41 kcal/mol was used as a positive control. As shown in **Table S3**, the scores of dFdCTP and lapatinib was closest to that of verapamil. The panoramic and detailed interactions between the ABCB1 model and the binding site of lapatinib and dFdCTP were shown in **Figure 5G**. A two-dimensional interactive map is depicted in **Figures S3A, B**. As revealed in **Table S4** and **Figures S3C, D**, comparative analysis of common amino acids involved at the substrate-binding sites between dFdCTP and lapatinib suggests that 8 out of the 16 are the same amino acid residues as those involved in dFdCTP interaction. Overall, these results indicate that dFdCTP is potentially the substrate of ABCB1.Since dFdCTP is the most critical agent for gemcitabine-induced apoptosis (42), it may be the reason that the upregulation of ABCB1 induces the drug resistance of tumor cells. Lapatinib interacts with the binding site of ABCB1 with a score of –8.93 kcal/mol, a score that is even higher than that of verapamil, suggesting that lapatinib possesses excellent affinities to ABCB1 and similarly may also bind competitively with the amino acid residues involved in dFdCTP interactions.

The ABCB1 transporter function has previously been reported to rely on energy from ATP catalyzed by the ABCB1-associated ATPase (43), which can be either stimulated or inhibited by ABCB1 substrates. We subsequently determined the effect of various concentrations of dFdCTP and lapatinib based on ABCB1 ATPase activity. Verapamil was set as the positive control. As exemplified in **Figures 5H–I**, both dFdCTP and lapatinib can stimulate the activity of the ABCB1, while the ability of lapatinib to produce ABCB1 activity is much stronger and more comparable to that of verapamil. This further confirms that both dFdCTP and lapatinib were substrates of ABCB1. Compared with dFdCTP, lapatinib is better at activating ABCB1 ATPases and similarly to verapamil, may bind with ABCB1 competitively.

We further investigated ability of lapatinib to decrease the efflux of ABCB1 substrates. After incubating with Rhodamine 123, one of the classical fluorescent substrates of ABCB1, FRH0201-Gem was then treated with various concentrations of lapatinib. Compared with cells stained with rhodamine without being treated with lapatinib (0 μM lapatinib),various concentrations of lapatinib were found to increase the mean fluorescence intensity of rhodamine in FRH0201-Gem cells in a concentration-dependent manner (**Figure 5J**). Additionally, incubating CC6062 with lapatinib for 30 minutes blocked ABCB1 activity and caused the accumulation of Rhodamine 123 within the cell, while Rhodamine 123 was ejected into the lumen of organoids in the control group (**Figure 5K**).

### Lapatinib Decreases the Efflux of dFdCTP From Cells by LC-MS/MS Analysis

After previously confirming dFdCTP as the substrate of ABCB1 and its ability to block the transportation of substrate of ABCB1, we further determined the direct effect of lapatinib on the efflux of dFdCTP, the results of which can confirm the synergistic effects of a combined lapatinib and gemcitabine treatment. FRH-0201 and FRH0201-Gem were treated with 1μM gemcitabine combined with various concentrations of lapatinib for 6 hours and then cells were collected for LC-MS/MS analysis and verapamil was set as the positive control. From the MS/MS spectrum of dFdCTP standards sample, we chose 504.2→326.4 as MRM ion pair and the peak appeared at a retention time of 0.68 min (**Figure 6A**). Then we assessed linearity using serially diluted working solutions with different concentrations, and the linearity of the calibration curve was well accepted with the correlation coefficients (R^2^) equal to 0.9971 and the range was from 10 to 1000 ng/mL (**Figure 6B**). The method accuracy (recovery rate) and precision (QC RSD) were all acceptable for the precise assessment of intracellular dFdCTP (**Figure 6C**). As shown in **Figure 6D**, the initial amount of dFdCTP found within FRH0201-Gem cells was less than that of FRH-0201 cells. This supports the idea that gemcitabine chemoresistant cells have a higher intracellular efflux due to the upregulation of ABCB1. We set verapamil as positive control and verapamil is proved to be able to inhibit the efflux of dFdCTP (**Figure 6D**). After a treatment with varying concentrations of lapatinib between 1 to 20μM, we detected that lapatinib promotes an accumulation of dFdCTP in both FRH-0201 and FRH0201-Gem comparable to that of not being treated with lapatinib (0μM Lapa) in a dose-dependent manner. This confirms the suppression of intracellular dFdCTP expulsion by lapatinib (**Figure 6D**).

## Discussion

As mentioned previously, a gemcitabine-based chemotherapy is considered to be the standard first-line treatment for advanced CCA cases, as supported by the results from several phase two and three clinical studies (6, 44, 45). However, because only a small portion of patients with CCA exhibit sensitivity and the median progression-free survival (PFS) is only about 5.3-7.7 months for those receiving gemcitabine-based chemotherapy (46, 47); initial and acquired drug resistance often results in recurrence, ultimately preventing it from being used extensively. One of the most common mechanisms found in the multi-drug resistance of CCA is the induction of drug efflux through ABC pumps (8, 48).

Our study suggests that dFdCTP, the active metabolite of gemcitabine, is the substrate of ABCB1, having proven to be capable of interacting with the binding pocket of ABCB1 and stimulating ABCB1-associated ATPase activity. It acts as the substrate of ABCB1-like topotecan and 5-fluorouracil. We have found that an increase in ABCB1 expression results in a parallel increase in efflux of dFdCTP which accounts for the prolific instances of drug resistance occurring after gemcitabine treatment. As such, new developments of targeted therapies and chemosensitizers, such as those targeting ABCB1 efflux pumps are crucial for improving future treatment options for cholangiocarcinoma patients.

The “Precision Medicine” revolution in the treatment of CCA is in the midst of rapid development due to improved knowledge in the molecular biology of these neoplasms. HER2 amplifications are among the most frequently targetable genetic variation found in eCCA, and can potentially be targeted with tyrosine kinase inhibitors (TKIs), such as lapatinib (49). Recent studies demonstrate that HER2 overexpression represents an independent prognostic factor for disease recurrence in CCA (26). Similarly, our research also suggests that HER2-overexpression positively correlates with poor prognosis in certain patients. Since HER2 overexpression represents an independent prognostic factor for prognosis in CCA and can be a therapeutic target, it is worthy of further investigation. A thorough perusal of scientific literature led us to find out about the efficacy of trastuzumab and pertuzumab in patients with HER2-positive CCA. Furthermore, we also found that HER2-targeted therapy includes not only monoclonal antibodies such as trastuzumab, but also tyrosine kinase inhibitors. In this study, we selected lapatinib (a type of TKI) for investigation. Our study demonstrates that HER2-overexpressed CCA is sensitive to lapatinib treatment *in vitro*. Lapatinib is the earliest developed TKI, a reversible inhibitor against both EGFR and HER2 targets. The further studies evaluating the efficacy of new generations of TKIs such as neratinib and tucatinib in HER2-positive cholangiocarcinoma is also something worth looking forward to. In addition, in our study while the cell lines FRH0201 and CC6062 were both found to be HER2 overexpressed, their IC50 values very different, the lapatinib IC50 value of FRH0201 was higher than the maximum plasma concentration (Cmax) achieved by the clinical dose (50). We believe that this may be caused by the presence of heterogeneity in CCA, which suggests that the responsiveness of HER2-overexpressing CCA to lapatinib may also be different, which is worth exploring further.

Organoids derived from primary tumor tissue make it possible to test and develop therapeutic approaches in a pre-clinical setting. It has been previously reported that the HER2 status can be maintained in organoids derived from breast cancer tumors. These HER2-positive breast cancer organoids were confirmed to be sensitive to drugs that inhibit the HER signaling pathway (51). Based on this, we established the first organoid model used to evaluate the inhibitory effects of lapatinib on HER2-overexpressed cholangiocarcinoma. As confirmed through our research, HER2 positive patient-derived tumor resections were similarly able to retain HER2 expression when cultured as organoids. Lapatinib was found to significantly suppress the growth of HER2- overexpressed CCA organoids. Since *in-vitro* cultured organoids often accurately correspond with responses found *in-vivo (* 52–56), we hypothesized that lapatinib could potentially also be effective when used in treating HER2- overexpressed CCA patients. This hypothesis still needs to be thoroughly examined through further testing with animal and patient-based clinical research.

Our research found that a combination of lapatinib and gemcitabine has a robust synergistic effect, having obtained CI values of less than 1 in both drug combination assays conducted on HER2-positive organoids and cell lines. Lapatinib has an affinity with ABCB1 that is comparable with that of verapamil, a well-known ABCB1 inhibitor. The ability of ABCB1 to expel its substrates relies on ATP hydrolysis by ATPases (57, 58). Based on this, we conducted an ABCB1-associated ATPase assay and found that lapatinib could stimulate ATPase activity in a concentration dependent manner. Additionally, lapatinib was also able to significantly increase the intracellular accumulation of Rhodamine 123, a fluorescent substrate of ABCB1. A LC-MS/MS analysis of dFdCTP in the presence of a concentration gradient of lapatinib provided evidence that lapatinib suppresses the efflux of dFdCTP and thus increases the cytoxic effect dFdCTP has on CCA cells. Our results indicate that lapatinib exerts a dual effect on HER2-overexpressed CCA, suppressing the growth of CCA cells by inhibiting HER2 and HER2-dependent downstream signaling pathways. Lapatinib is also found to be able to bind to ABCB1 competitively, increasing gemcitabine efficacy by blocking the efflux of dFdCTP from tumor cells, and promoting its accumulation within cells. A combination of lapatinib and chemotherapy has previously been proven to be safe and efficient for HER2-overexpressed gastroesophageal adenocarcinoma and breast cancer (59, 60). As such our study provides clear evidence that lapatinib can be substantially coordinated with gemcitabine as a first line treatment for HER2-overexpressed CCA cases. We have provided an excellent preclinical evaluation of the efficacy of a lapatinib-gemcitabine combination-based therapy which can be applied to future treatments of HER2-overexpressed cholangiocarcinoma, while simultaneously shedding new light on the potential of molecular targeted CCA therapies.

## Conclusion

Lapatinib inhibits tumor growth in organoids and cell lines overexpressing HER2 and circumvents ABCB1-mediated chemoresistance after gemcitabine treatment. We believe that this is a precursor for further clinical investigation of the effectiveness of a lapatinib-gemcitabine combined treatment in HER2-overexpressed cholangiocarcinoma.

## Data Availability Statement

The original contributions presented in the study are included in the article/**Supplementary Material**. Further inquiries can be directed to the corresponding authors.

## Ethics Statement

The studies involving human participants were reviewed and approved by Ethics Committee of Beijing Tsinghua Changgung Hospital. The patients/participants provided their written informed consent to participate in this study.

## Author Contributions

ZB and ZG contributed equally to conception and design of the study. JL, LH, and QL performed the statistical analysis. ZB and Y-AC wrote the first draft of the manuscript. PZ, Y-AC, ZG, and JL wrote sections of the manuscript. JD and YW designed and supervised the entire project. All authors contributed to the article and approved the submitted version.

## Funding

This work was supported by CAMS Innovation Fund for Medical Sciences (2019-I2M-5-056), National Natural Science Foundation of China (No. 81930119, 82090050), Beijing Hospitals Authority’ Ascent Plan (DFL20190901), and Tsinghua University Spring Breeze Fund (2021Z99CFZ008).

## Conflict of Interest

The authors declare that the research was conducted in the absence of any commercial or financial relationships that could be construed as a potential conflict of interest.

## Publisher’s Note

All claims expressed in this article are solely those of the authors and do not necessarily represent those of their affiliated organizations, or those of the publisher, the editors and the reviewers. Any product that may be evaluated in this article, or claim that may be made by its manufacturer, is not guaranteed or endorsed by the publisher.

## Supplementary Material

Supplementary Figure 1 HER2 amplification/overexpression profiles within CCA patient populations. **(A)** HER2 amplification profiles in biliary tract carcinoma as analyzed by cBioPortal. **(B)** Overall survival (OS) curve analysis of patients diagnosed as either HER2 positive or HER2 negative. **(C)** Images of HER2 immunohistochemistry in CCA tissue CC6062. **(D)** Images of HER2 FISH in CCA tissue CC6062.

Supplementary Figure 2 A detailed distribution of G1 cycle arrested and apoptotic cells induced by lapatinib (Lapa), gemcitabine (Gem) or a combination (Lapa+Gem) treatment in FRH-0201. **(A)** Cell cycle arrest was induced by lapatinib, gemcitabine or a combination of both compounds for a duration of 48 h. **(B)** Apoptosis was induced by lapatinib,gemcitabine or combination of both compounds for a duration of 48 h. **(C)** Apoptosis and cell cycle arrest in FRH-0201 was measured after being treated with lapatinib (Lapa) for the above indicated time periods.

Supplementary Figure 3 Lapatinib and gemcitabine show a strong binding affinity towards ABCB1. **(A)** A two-dimensional diagram of the binding of lapatinib with ABCB1. **(B)** A two-dimensional diagram of the binding of dFdCTP with ABCB1. In both **(A, B)** aromatic residues are colored in green, whereas polar amino acids are shown in magenta. **(C)** A Venn diagrammatic representation of amino acid residues shared by dFdCTP, verapamil, and lapatinib at the substrate-binding domain of ABCB1. **(D)** Detailed depiction of dFdCTP and lapatinib interactions with ABCB1 binding pockets. The dFdCTP molecule is represented using a sphere and pole model and covered with a solid-gray pocket, while the lapatinib molecule is represented using a pole model and covered with a dotted-green pocket.

Supplementary Figure 4 EGFR status of cells and the inhibitory effect of Cetuximab (CTX) on FRH0201. **(A)** EGFR IHC staining of tissue and organoid revealed that both CC6062 and CC9630 were EGFR-negative, while CC2196 was positive. Scale bar= 100 μm **(B)** EGFR protein expression in CCA cell lines was detected by Western blotting. **(C)** Both cetuximab (CTX) and lapatinib (Lapa) were able to suppress p-EGFR of FRH-0201. **(D)** Growth inhibitory effect curves of cetuximab (CTX) in FRH-0201 proved that FRH-0201 is resistant to cetuximab (CTX).

Supplementary Figure 5 Establishment of Gemcitabine-resistant FRH0201-Gem. **(A)** Bright-field image of FRH-0201 and FRH0201-Gem. Scale bar= 500 μm. **(B)** Parental FRH-0201 and gemcitabine-resistant FRH0201-Gem were plated in six well plates and cells were allowed 48 h to initiate colonies. Then cells were incubated at 5 μM gemcitabine for an additional 12 days to assess the drug resistance of FRH0201-Gem using colony-forming assay. **(C)** Colony assay results for FRH-0201 and FRH0201-Gem treated with gemcitabine treatments.

Supplementary Video 1 Time-lapse movie of organoids treated with DMSO(Control), 5 μM gemcitabine (Gem), 5 μM lapatinib (Lapa), and 5 μM gemcitabine+5 μM lapatinib (Gem+Lapa). The movie was initiated as the drug was added into the medium and continued for 4 days. Each picture was taken at an interval of 1 hour using the Live Cell Imaging System (Etaluma, Ameirica). **(A–D)** CC6062 treated with DMSO, Lapa, Gem and Gem+Lapa. **(E, F)** CC2196 treated with DMSO and Lapa. **(G, H)** CC9630 treated with DMSO and Lapa.
