# Supplementary figures and images for: Lapatinib Suppresses HER2-Overexpressed Cholangiocarcinoma and Overcomes ABCB1– Mediated Gemcitabine Chemoresistance
Source: Front Oncol. 2022 Apr 8;12:860339. doi: 10.3389/fonc.2022.860339 (PMC9033256; doi:10.3389/fonc.2022.860339)

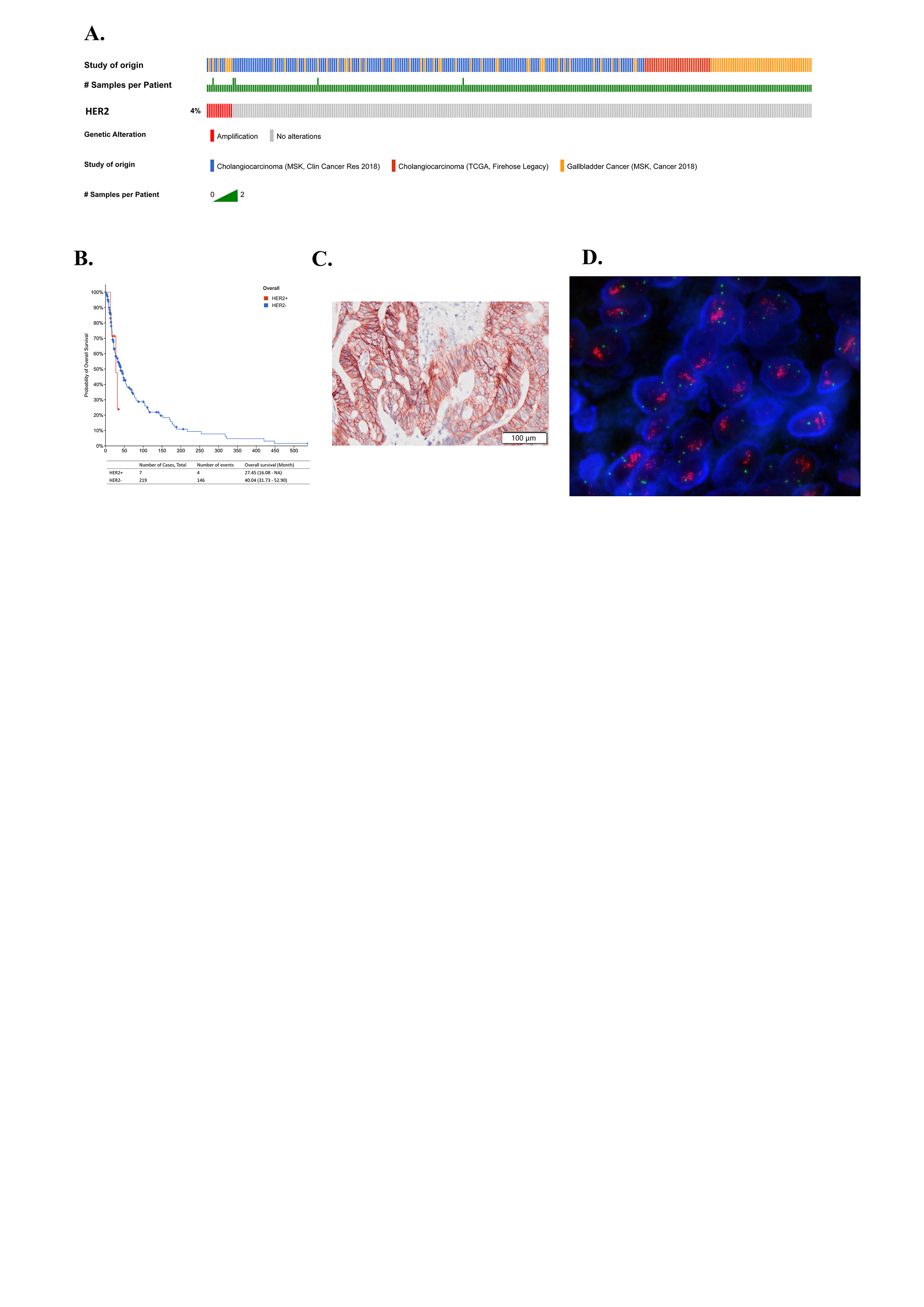

Supplement: Supplementary Figure 1 — HER2 amplification/overexpression profiles within CCA patient populations. (A) HER2 amplification profiles in biliary tract carcinoma as analyzed by cBioPortal. (B) Overall survival (OS) curve analysis of patients diagnosed as either HER2 positive or HER2 negative. (C) Images of HER2 immunohistochemistry in CCA tissue CC6062. (D) Images of HER2 FISH in CCA tissue CC6062. [file Image_1.tif]

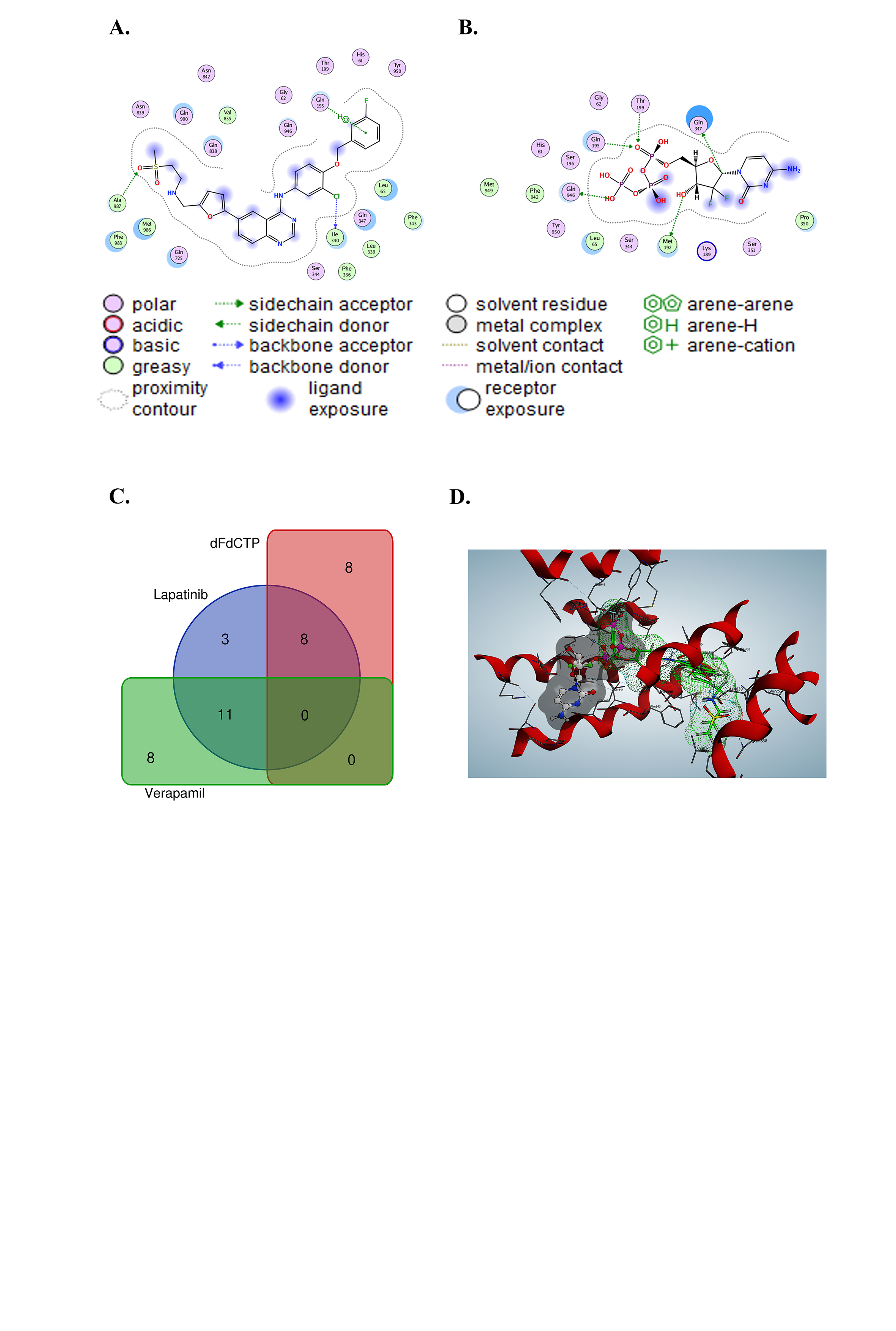

Supplement: Supplementary Figure 3 — Lapatinib and gemcitabine show a strong binding affinity towards ABCB1. (A) A two-dimensional diagram of the binding of lapatinib with ABCB1. (B) A two-dimensional diagram of the binding of dFdCTP with ABCB1. In both (A, B) aromatic residues are colored in green, whereas polar amino acids are shown in magenta. (C) A Venn diagrammatic representation of amino acid residues shared by dFdCTP, verapamil, and lapatinib at the substrate-binding domain of ABCB1. (D) Detailed depiction of dFdCTP and lapatinib interactions with ABCB1 binding pockets. The dFdCTP molecule is represented using a sphere and pole model and covered with a solid-gray pocket, while the lapatinib molecule is represented using a pole model and covered with a dotted-green pocket. [file Image_3.tif]

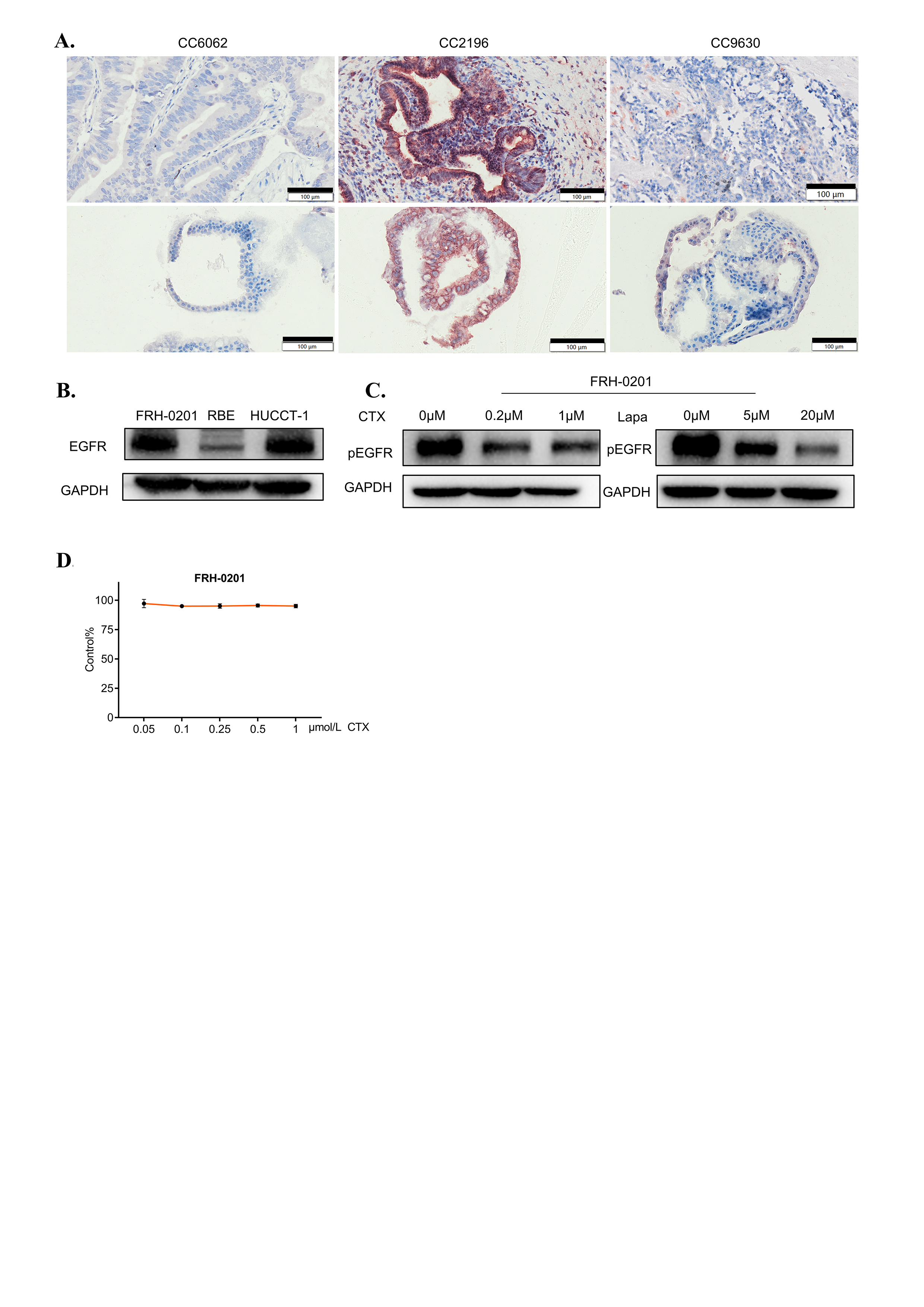

Supplement: Supplementary Figure 4 — EGFR status of cells and the inhibitory effect of Cetuximab (CTX) on FRH0201. (A) EGFR IHC staining of tissue and organoid revealed that both CC6062 and CC9630 were EGFR-negative, while CC2196 was positive. Scale bar= 100 μm (B) EGFR protein expression in CCA cell lines was detected by Western blotting. (C) Both cetuximab (CTX) and lapatinib (Lapa) were able to suppress p-EGFR of FRH-0201. (D) Growth inhibitory effect curves of cetuximab (CTX) in FRH-0201 proved that FRH-0201 is resistant to cetuximab (CTX). [file Image_4.tif]

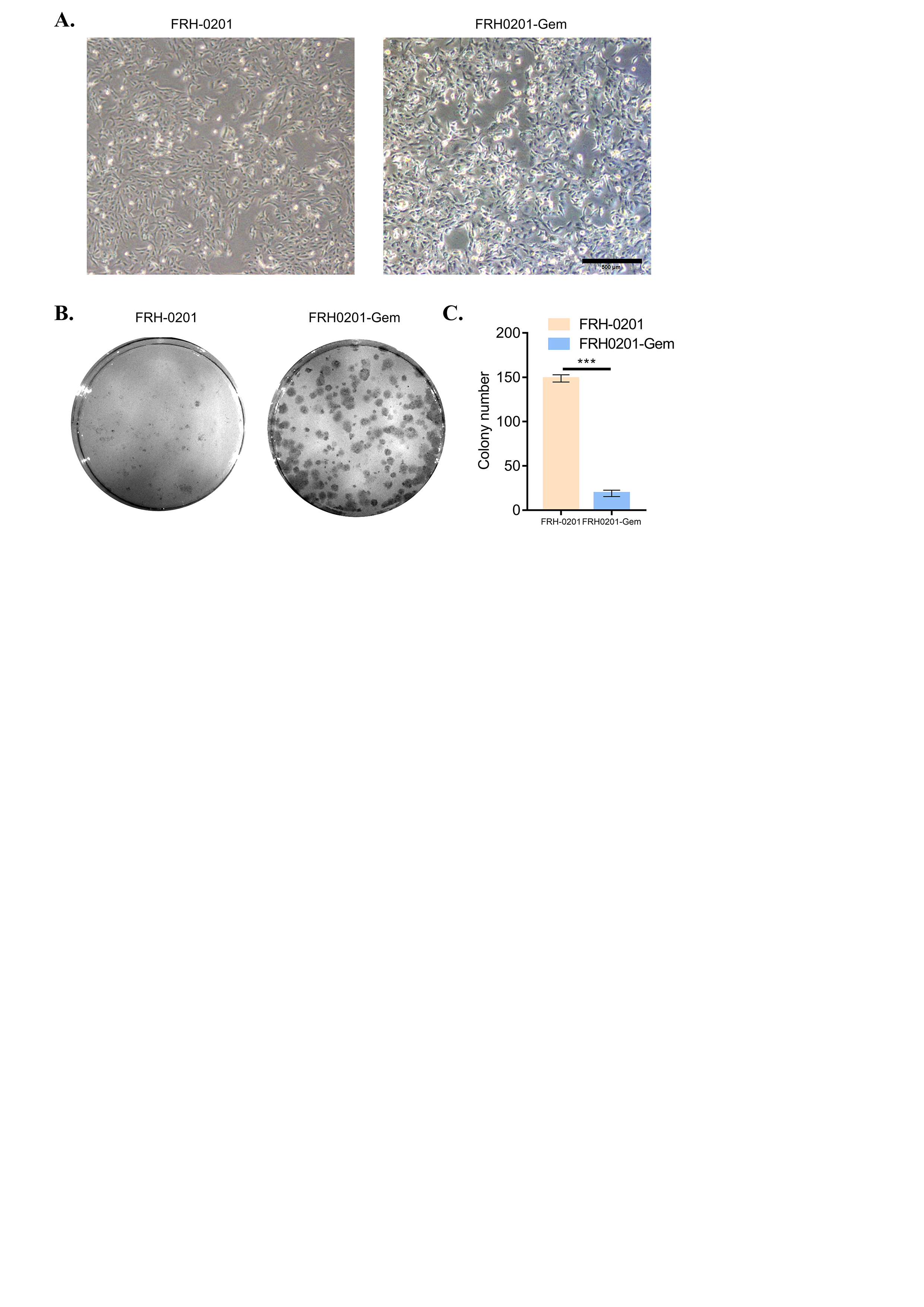

Supplement: Supplementary Figure 5 — Establishment of Gemcitabine-resistant FRH0201-Gem. (A) Bright-field image of FRH-0201 and FRH0201-Gem. Scale bar= 500 μm. (B) Parental FRH-0201 and gemcitabine-resistant FRH0201-Gem were plated in six well plates and cells were allowed 48 h to initiate colonies. Then cells were incubated at 5 μM gemcitabine for an additional 12 days to assess the drug resistance of FRH0201-Gem using colony-forming assay. (C) Colony assay results for FRH-0201 and FRH0201-Gem treated with gemcitabine treatments. [file Image_5.tif]
